# Odor Representations in the Rat Olfactory Bulb Change Smoothly with Morphing Stimuli

**DOI:** 10.1016/j.neuron.2008.01.008

**Published:** 2008-02-28

**Authors:** Adil G. Khan, Mukund Thattai, Upinder S. Bhalla

**Affiliations:** 1 National Centre for Biological Sciences, Tata Institute of Fundamental Research, Bellary Road, Bangalore 560065, India

**Keywords:** SYSNEURO

## Abstract

Many species of mammals are very good at categorizing odors. One model for how this is achieved involves the formation of “attractor” states in the olfactory processing pathway, which converge to stable representations for the odor. We analyzed the responses of rat olfactory bulb mitral/tufted (M/T) cells using stimuli “morphing” from one odor to another through intermediate mixtures. We then developed a phenomenological model for the representation of odors and mixtures by M/T cells and show that >80% of odorant responses to different concentrations and mixtures can be expressed in terms of smoothly summing responses to air and the two pure odorants. Furthermore, the model successfully predicts M/T cell responses to odor mixtures when respiration dependence is eliminated. Thus, odor mixtures are represented in the bulb through summation of components, rather than distinct attractor states. We suggest that our olfactory coding model captures many aspects of single and mixed odor representation in M/T cells.

## Introduction

Attractor networks are the most common models for explaining memory storage and recall, and input-output transformations in networks of neurons (Amit, 1989; Hopfield, 1982; Rolls and Treves, 1998). These Artificial Neural Networks (ANNs) have multiple stable states. Each such state is a specific, stable pattern of spatial and possibly temporal activity across the network. The key attribute of such an attractor network is that when its neurons are stimulated with patterned input, the ANN converges to the stored pattern most closely resembling the input.

A large body of work on categorical perception (Rotshtein et al., 2005; Wyttenbach et al., 1996) might be explained by such attractor-based models. The few explicit tests of these ideas have provided some evidence for signatures of attractor dynamics in different systems (Freedman et al., 2001; Guzowski et al., 2004; K. Jezek et al., 2006, FENS Forum, abstract; Lee et al., 2004; Leutgeb et al., 2005; Vazdarjanova and Guzowski, 2004; Wills et al., 2005). The recent work by Wills et al. and Jezek et al. in the hippocampus and entorhinal cortex provides striking results in favor of such theories.

It has been proposed that the mammalian olfactory bulb (OB) may also act as an attractor-based neural network (Hendin et al., 1998). A directly testable prediction of such a model is that the network should transition abruptly from one stable state to another when it is presented with a stimulus set that progresses from one distinct odor stimulus to another through a series of intermediate mixtures. A related proposal, based on experiments and models, suggests that odor representations in the OB take the form of chaotic attractors (Freeman, 1991; Freeman and Grajski, 1987). This idea is based on EEG recordings, and is therefore not directly comparable to our single-unit recordings, but it too predicts abrupt transitions between responses to different odorants.

To address the question of whether olfactory responses change abruptly, one must first consider how odors are represented in the OB. This is necessary to quantify transitions between olfactory representations. These representations are comprised of OB cell activity patterns in response to an odor stimulus, and are transmitted to downstream regions through the principal output neurons, the mitral/tufted (M/T) cells. The activity of individual M/T cells can be patterned over respiration cycles, both in terms of their baseline activity and in terms of their response to an odor (Bhalla and Bower, 1997; Chaput and Holley, 1980; Macrides and Chorover, 1972). This patterned activity is preserved in cells downstream in the piriform cortex (N. Uchida and Z.F. Mainen, 2006, Soc. Neurosci., abstract; Wilson, 1998) and hippocampus (Deshmukh and Bhalla, 2003) and may be used for encoding olfactory information. Different odors can evoke distinct patterns, which are often complicated combinations of excitation and inhibition.

There is evidence that respiration-patterned activity is primarily driven by olfactory receptor neuron (ORN) input patterns (Sobel and Tank, 1993). Patterned activity to air alone may be explained by recent work on mechanosensitive properties of ORNs (Grosmaitre et al., 2007). Studies have suggested that these patterns are shaped further by processing within the bulb, through the interaction of glomerular and M/T cell activity with inhibitory neurons like the periglomerular and granule cells (Li and Hertz, 2000; Linster and Cleland, 2004; Linster and Hasselmo, 1997; Shepherd, 2003). There have been many studies attempting to provide a characterization of this behavior (Cang and Isaacson, 2003; Chalansonnet and Chaput, 1998; Giraudet et al., 2002; Hamilton and Kauer, 1989; Harrison and Scott, 1986; Meredith, 1986; Motokizawa, 1996; Wellis et al., 1989). Nevertheless, these studies do not establish a unified model to explain how the patterned responses of M/T cells can encode both identity and intensity of odors. The issue of odor combinations has been addressed in human studies (Laing et al., 1984) and in many systems with nonrespiration-based odor sampling (Broome et al., 2006; Kang and Caprio, 1995; Tabor et al., 2004). Only one study has addressed odor mixtures in the context of respiration-patterned responses (Giraudet et al., 2002), though here too this issue has not been incorporated into a unified model.

Our experiments were designed to answer the initial question: Does the OB show signatures of attractor dynamics? In the process we have addressed the fundamental issue of the representation of odor identity and intensity in M/T cells, including their responses to varying odor mixtures. This led us to formulate a unified model explaining the behavior of these cells along multiple dimensions of odor identity, intensity, and combinations in a limited concentration range.

## Results

### Recordings and Respiration-Tuned Responses

**Figure 1:**
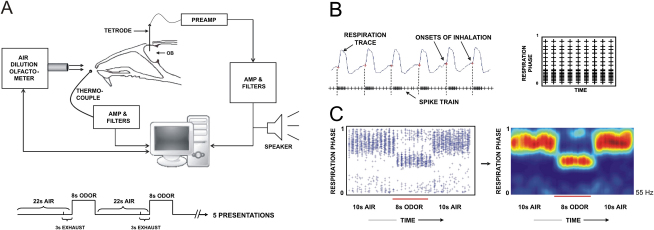
Recording Procedures and Data Representation (A) Extracellular single-unit recordings were made using tetrodes, and the signal was amplified and filtered and acquired on a computer. The respiration of the rat was typically steady at 1 Hz and was monitored with a thermocouple placed in front of its nostril. The odor presentation protocol is shown below. (B) A schematic of the construction of a respiration raster that shows the respiration-locked firing pattern of a cell. The spike train is divided into each respiration cycle, and each spike is replotted with the respiration phase on the y axis and time of respiration cycle start (or simply time) on the x axis, aligned to the odor valve onset. (C) Data from a cell with five trials of an odor presentation superimposed. Each + sign represents an action potential. The color plot to the right is the same data after smoothing and color coding. The red bar indicates the duration of the odor stimulus here and in subsequent figures. This cell responds to the odor by changing its respiration tuning, but not its firing rate. The frequency shown on the side of the plot is the maximum firing rate on this figure here and in subsequent plots.

We characterized responses of rat OB M/T cells to different odors and analyzed how these responses changed when the stimulus was “morphed” from one odor to another through a series of intermediate mixtures. In order to do this, we performed extracellular single-unit recordings from anesthetized, freely breathing rats, using tetrodes. We recorded from 593 M/T cells in these data sets and have analyzed a subset based on stability and responsiveness to one or more odors. We simultaneously recorded the breathing of the rat through a thermocouple placed in front of its nostril. The anesthetized rats typically respired at a steady rate of 1 Hz. We delivered controlled pulses of odor stimuli to the rats' noses using an air-dilution olfactometer (Figures 1A and 1B; see Experimental Procedures).

In our recordings we frequently observed a modulation of M/T cell firing rate over the respiration cycle, as previously reported (Deshmukh and Bhalla, 2003; Macrides and Chorover, 1972). We refer to this phenomenon as respiration tuning. Figure 1B is a schematic description of our procedure for characterizing respiration tuning, and Figure 1C shows an example of a cell with five trials of odor presentation overlaid. This “respiration raster” was smoothed and color-coded for visualization (see Experimental Procedures). In this example, without any odor, the cell fired preferentially in the later part of the respiration cycle. In the presence of odor, the cell responded by changing its respiration tuning pattern rather than its mean firing rate. We used this color-coded representation of an overlay of five trials in several of our following illustrations.

### Odor Response Distributions

We first tested each cell with 1% dilution of saturated odor vapor from our panel of four odors: iso-amyl acetate, methyl amyl ketone, 1,4-cineole and (+) limonene (at least three odors were tested in the naive rats and the two familiar odors in the familiarized rats—see below). The fraction of our total set of neurons responding to at least one odorant was 50% in our study, in line with published data (Giraudet et al., 2002). Out of these, identical responses to two or more odorants were relatively common (23% of total), while different responses to two odors were rare in our study (8%, or 47/593, of which only 66% were stable through the length of the morph experiments). While our four-odor panel was small, all four odorants had very different structures and functional groups, and were designed to be a representative sampling of functionally significant responses.

### Intermediate Responses to Mixtures

We looked for cells that showed different respiration-tuned responses to two odors. On finding such a cell, we performed the morph protocol, i.e., presented mixtures of the two odors (labeled A and B) with the following compositions: [1.0A 0.0B], [0.8A 0.2B], [0.6A 0.4B], [0.4A 0.6B], [0.2A 0.8B], [0.0A 1.0B]. We refer to this as the “morph sequence.” Each sequence took approximately 30 min. When the recordings were exceptionally stable, we were able to perform the morph sequence in both directions, and if possible a second time each.

Since most theoretical models of attractor-based networks rely on the network being trained on the stimuli to be stored (Hertz et al., 1991), we familiarized one group of rats on one pair of odors (I and M) for 5–8 days before the recording (see Experimental Procedures). We compared these responses with those from naive rats. We found no difference between the two groups in all subsequent analysis and therefore pooled all the cells (n = 32 cells; 19 naive, 13 odor-familiarized; distribution in categories is not different for the two groups, chi-square test, p < 0.05; see Table S1 available online).

**Figure 2:**
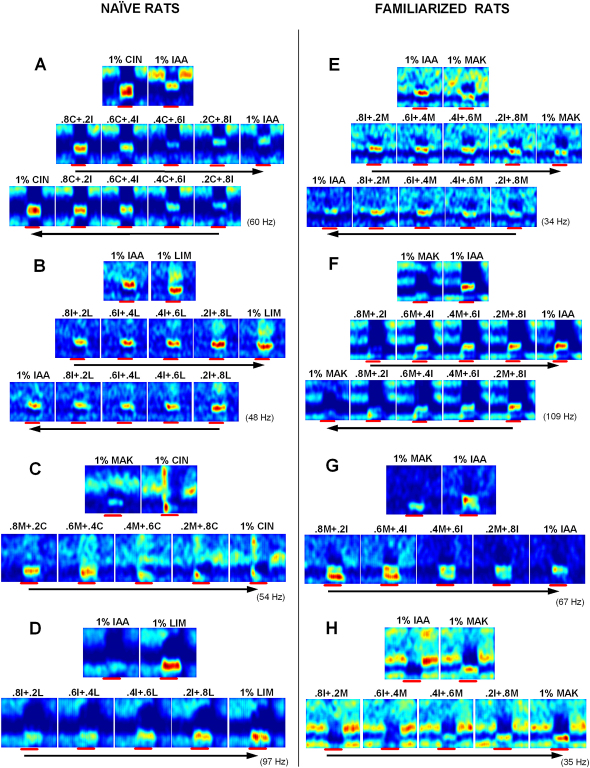
Odor Responses Pass through Intermediates During Exposure to the Morph Sequence (A)–(D) show cells from naive rats. (E)–(H) are from rats that were familiarized with the odors for 5–8 days. Each panel is from a cell that responded to two odors differently, and these two responses are shown on the top of each panel. Below these are the responses to the mixtures of the two odors in the morph sequence, which can be seen to pass through intermediate forms of responses. In a few particularly stable recordings, morph sequences were recorded in both directions as in (A), (B), (E), and (F). Arrows indicate temporal order of recordings. The color coding is the same for all plots in each panel. Numbers in brackets are the highest firing rate in each panel.

In Figure 2 we present some examples of the neuronal responses to different pure odors and the morph sequence(s) between them. Our findings were the following: (1) cells could display clearly distinct responses to different odors; (2) the responses to intermediate mixtures were intermediate between the two pure odor cases; and (3) the baseline activity of the cell drifted to varying degrees, as measured by the firing rate and respiration tuning in the air periods of the same cell. The response to the same odor after an interval also drifted. This drift could be due to anesthesia effects (see Discussion) and has been included in our quantification of the noise in the system (see Supplementary Material). However, despite this drift, the responses varied smoothly between the two pure odor cases.

One of two prominent ways a cell behaved to a morph sequence was with a “band” of excitation gradually shifting along the respiration phase axis. For example, in Figure 2A, the response to 1% cineole (odor-on: red bar) is a large shift in respiration tuning with a broad band of excitation, and for 1% iso-amyl acetate, it is a smaller shift in respiration tuning and a narrower band. The morph protocol for this cell was done in both directions, and one can observe the band gradually shifting higher and becoming narrower in the forward morph while the reverse occurs in the reverse morph. Other instances of such shifting bands are shown in Figures 2B and 2E.

The second type of prominent behavior was firing rate building up or fading out in specific phases of respiration. This can be seen in Figures 2B, 2D, and 2F–2H.

A further class of responses is shown in Figure 2C, which showed firing patterns changing even over the course of the 8 s odor presentation. We could not use this cell (n = 1) for subsequent analysis, since that assumes a single stable respiration tuning pattern for each odor. However, the basic result, smooth transitions of responses, is still apparent in this example.

**Figure 3:**
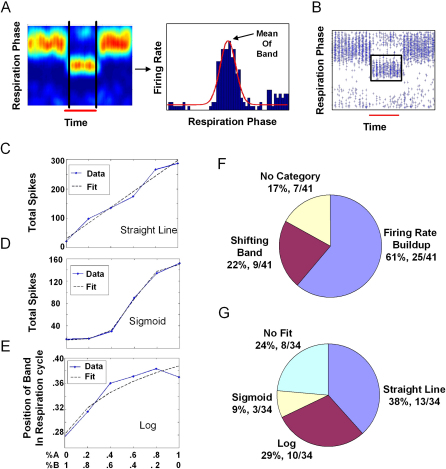
Simple Analysis of Morphing Responses (A) Estimating peak firing phase using Gaussian fit. (B) Firing rate buildup in a defined range of respiration phase. Rates were calculated by summing all spikes within the selected respiration phase range, shown as a box on the raster plot. (C) Example of change of response as a function of composition of the mixture fitting a straight line. (D) Sigmoid response. (E) Log response. (C) and (D) are from cells with firing rate buildup and (E) is from a cell with a shifting band. (F) The distribution of the 41 morphs from 32 cells between firing phase and firing rate calculations. (G) The distribution of cells between straight line, log, sigmoid, and other categories.

As a first-pass quantification, we extracted the values of the two features mentioned above for each morph experiment (41 morphs from 32 cells): the position of the band or the firing rate in a specific phase of respiration. For cells which had both these effects, we chose the more prominent one. Some cells could not be categorized in either group. The distribution to these groups is shown in Figure 3F.

To estimate the position of the band, we fitted Gaussians to the binned data (Figure 3A, see Experimental Procedures). As a measure of firing rate, we summed up the total number of spikes in a box enclosing the excitatory band (Figure 3B). We plotted these values against the composition of the mixture. These curves were fit to straight lines, logarithms, or sigmoids. Examples are shown of cells which were best fit with straight lines (Figure 3C), sigmoidal curves (Figure 3D), or logarithmic curves (Figure 3E) (p < 0.01 and best explained variance by the F statistic). An abrupt transition from one stable attractor to another would be expected to give a steep sigmoid-like curve. As can be seen in the distribution in Figure 3G, all three categories existed in these morph sequences. Further, most morph sequences belonged to the straight line (38%) or log (29%) categories (see Experimental Procedures). Therefore this preliminary analysis argues against strong attractor dynamics in OB responses to odorants.

Though we had recordings of some morph sequences repeated either in the reverse (n = 6) or forward (n = 1) direction, we did not have sufficient data to analyze effects of hysteresis.

These initial findings were suggestive, but were based on a model-free analysis that did not provide a deeper explanation of why the parameters we tracked over the morph sequence were the relevant ones. This analysis also failed to explain the following observations: (1) the presence of cells from which simple features could not be extracted; (2) cells in which multiple features coexisted and changed at different rates (such as in Figure 2B with two regions of excitation, and Figure 2H with excitation followed by inhibition); and (3) the exact specific shapes of the intermediate responses.

Thus, our preliminary analysis argued against strong attractor dynamics in the OB, but this analysis was limited in several ways. To overcome these limitations, we developed a more complete model of M/T cell responses as described below.

### The Model: Addition of Excitatory and Inhibitory Input Functions of Respiration Phase

**Figure 4:**
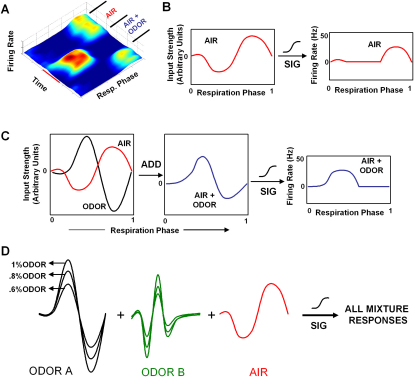
Schematic of Model (A) Example of a cell's response to an odor shown in three dimensions. (B) The air input strength function of the cell (left) is a function of the respiration cycle during air presentation. When passed through a sigmoid, the air input function results in the firing rate over the respiration cycle for the air period. (C) The odor input strength function (black) is added to the air input strength function (red) to give the function AIR + ODOR (blue). This AIR + ODOR function, when passed through the same sigmoid, will result in the AIR + ODOR firing rate function (right). (D) All possible mixture responses are obtained by scaling each odor input strength function and adding them to the air function. This is passed through the sigmoid to obtain the response of the cell to the mixture.

Here we describe a phenomenological model for M/T cell activity that explains most aspects of these complex responses to odors and odor mixtures. The model may be summarized as follows (Figure 4):

1. Odor input: Any odor that elicits a response from a cell provides an input that is a combination of excitation and inhibition as a function of the respiration phase (Figure 4C).
2. Air input: Similarly, air itself provides an input that is a combination of excitation and inhibition as a function of the respiration phase (Figure 4B).
3. Scaling: The odor input scales in amplitude, but not in shape, when odor intensity changes (Figure 4D).
4. Additivity: The weighted odor and air inputs sum to give the total input, which is also a function of respiration phase (Figure 4D).
5. Firing rate: The output of a cell, measured in terms of firing rate, is a sigmoidal function of its input (thus, strongly negative inputs give zero firing rate, while strongly positive inputs elicit the saturation firing rate). The summed inputs, when transformed through this sigmoid, give the instantaneous firing rate as a function of the respiration phase.

Using this model, we should be able to completely define the response to any odorant mixture given just the underlying air and pure odor inputs. We define an “input strength function” to be the input mentioned in point 1. It is a measure of the actual total input impinging on the cell from a source, the sum total of which, when passed through the abovementioned sigmoid, results in the observed firing rate of the cell.

In order to compare the predictions of this model with our experimental observations, we took two further steps.

First, the raw data were in the form of individual action potentials, while the model predicted instantaneous firing rates. In order to enable comparison, we transformed the observed firing events into firing rates as follows. We first separated the respiration raster along the time axis into two windows corresponding to the “Air” and “Air+Odor” epochs. Within each epoch, we then pooled the firing events into *N_R_* = 17 equally spaced bins along the respiration phase axis. The bin size was chosen to strike a balance between two competing effects: too large, and variations in firing rate over the respiration phase would be missed; too small, and Poisson fluctuations would produce large errors in the estimated rate. We found that using 10 bins instead of 17 reduced the quality of our fit, while using 25 bins instead of 17 left the quality essentially unchanged (see Figure S6 available online).

The observed responses to air and to odor were thus represented as two *N_R_*-dimensional vectors of instantaneous firing rates.

Second, the model gave us considerable freedom in choosing how the inputs to the cell were represented as functions of the respiration phase. One option was to represent the inputs as sums of positive and negative Gaussians, as is often done in center-surround models of spatial excitation and inhibition. However, in the absence of detailed mechanistic information, we had no reason to select this functional form over any other. A more natural choice, given that the response is periodic over the respiration cycle, would be to write the input as a truncated Fourier series. We repeated the complete analysis using 9 and 11 coefficient Fourier sums as input strength functions. These Fourier expansions did not follow some of the sharper changes during the respiration cycle, suggesting that even 11-coefficient series might be insufficient to represent the data. Furthermore, the resultant models did not explain 50% of the morph sequences. We opted for the simplest approach, in which the inputs themselves were represented, like the firing rate data, as *N_R_*-dimensional vectors running over the respiration phase. This makes no assumptions about functional form, but instead spans the space of all possible functions.

Although we did not use a compact representation of the odor or air inputs, the model was parsimonious. This was because we were able to capture the responses to *all* mixtures of odors in terms of just *three* input functions, one each for air, odor A, and odor B. More precisely: a typical experiment involved a morph sequence consisting of [1.0A 0.0B], [0.8A 0.2B], [0.6A 0.4B], [0.4A 0.6B], [0.2A 0.8B], [0.0A 1.0B], as well as exposure to air alone. These seven curves (functions of respiration phase) involved 7 × 17 = 119 datapoints. Our model uses just three of these curves (the responses to pure A, pure B, and air alone) to predict the four remaining mixture responses (4 × 17 = 68 datapoints) using just nine parameters, making this a highly constrained fit. In practice, we estimate the three pure responses as well as the remaining nine parameters simultaneously, so as to fit all seven curves; see Experimental Procedures.

Parameter estimation was carried out as follows. For any given experiment, we first represented the data as 17-bin vectors of firing rates, with one vector for each intermediate odor mixture in a morph sequence. Starting with an initial guess of parameter values, we then used the model to generate predicted firing rates for each intermediate odor mixture. We then quantified the error in terms of a chi-square statistic, essentially summing the squared deviations of predictions from observations. By iteratively minimizing this score, we finally obtained our best-fit parameters (Press and Teukolsky, 1992).

**Figure 5:**
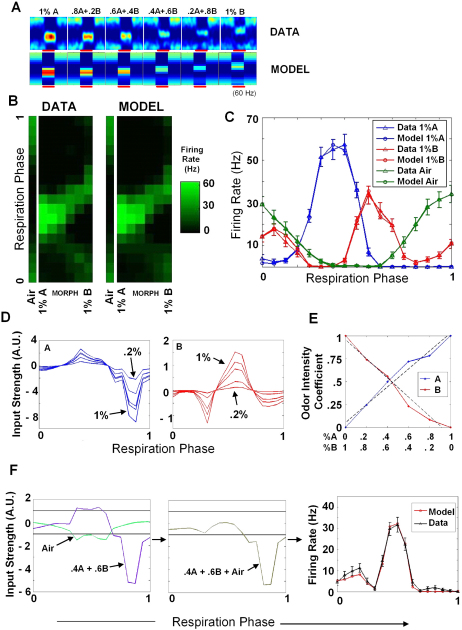
Model Validation and Predictions (A) Above is the original data showing odor responses to the morph sequence, and below is the model prediction. This is the same example from Figure 2A, reverse morph. Cineole is odor A and iso amyl acetate is odor B. (B) Alternate representation of data in (A) and its comparison with the model. Odor periods are placed alongside for each mixture of the morph sequence. The model representation is very close to the experiment. (C) Overlaid respiration phase versus firing rate plots for the data (with error bars) and the model. Curves are shown for air, 1%A, and 1%B. (D) Input strength functions for odors A and B, showing scaling with different odor concentrations. (E) Coefficients for odors A and B as a function of odor mixture. In this example the coefficients fit a straight line. (F) An illustration of the process of obtaining the mixture response of .4A + .6B. The two respective curves from (D) of .4A and .6B are added to obtain the purple curve (left). The air baseline is shown in green. The two horizontal lines correspond to the approximate lower and upper cutoffs imposed by the sigmoid, of zero and saturated firing, respectively. Adding the three curves gives the brown Air + .4A + .6B curve (center). This is passed through the sigmoid to give the red curve (right) showing the response for this particular mixture. It is a good fit to the experimental data, in black. Error bars indicate SEM.

This model was a good description of the data in 80% (33 out of 41) of the morph sequences that we obtained from the original data set of 32 cells (see below). An example of one cell is in Figure 5, which is the same cell from Figure 2A (odors are here labeled A and B for simplicity). Figure 5A compares the data to the results obtained from the model for one morph sequence. Figure 5B is the same comparison in a different format. As is clearly seen in both these panels, there were a number of features that changed over the morph sequence, and most of them were captured in the model. Figure 5C shows overlaid the data and model for the pure odors and air with error bars.

The underlying input strength functions for the two odors that emerged from this analysis are shown in Figure 5D. These are the functions of Figure 4D, the contributions of the odors on top of the air baseline. It is interesting to note that these functions have “inhibitory surrounds” around their excitatory components that account for the bands shifting in the morph sequence as opposed to the bands fading in and fading out.

The odor intensity coefficient is the scale factor by which the input strength function is multiplied when the odor is present at a particular concentration. It was defined as 1 for an odor at 1% concentration, and 0 when the odor was not present. The coefficients for intermediate odor concentrations were calculated by fitting them to the data as part of the process of computing the input strength functions. These odor intensity coefficients are a measure of the effect of the odor on the cell and are plotted in Figure 5E, against the externally applied odor concentrations. In this case, these plots were both best fit with a straight line. Figure 5F illustrates the process of addition of input strength functions for one of the mixtures (.4A + .6B), and also shows the approximate upper and lower cutoffs imposed by the sigmoid (horizontal black lines). The green air curve in the left panel gave the Model Air curve in Figure 5B when passed through the sigmoid. Similarly, the brown curve in the middle panel, when passed through this sigmoid, gave the red Model curve in the right panel, and this is overlaid with the data for this particular mixture of .4A + .6B.

### Validating the Model in Terms of Statistical Significance

**Figure 6:**
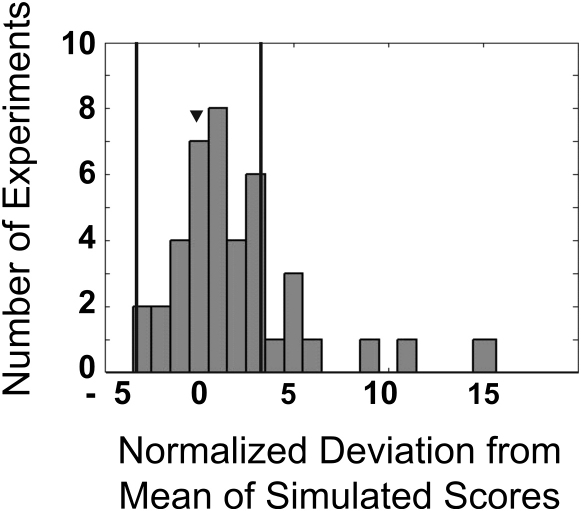
Population Data and Validation of the Model A distribution of simulated chi-square scores was calculated for each morph experiment in a Monte Carlo manner (n = 41 morphs). The difference between the mean of the simulated scores and the score from fitting the data, normalized by the standard deviation of the simulated score distribution, was found. The histograms of these values are plotted. The two vertical lines are at ±3.29. Eighty percent of morphs (33/41) were within this range. The arrowhead indicates the bin in which the example from Figure 5 belongs.

As our model included a large number of parameters and a sigmoidal nonlinearity, it was particularly important to employ rigorous tests for statistical significance. The first step in our evaluation was to understand sources of noise in the measurements. During a single-odor presentation session of five trials, measured firing rates displayed precisely the standard deviation expected from Poisson statistics (Figure S2). However, a comparison of results between different odor presentations revealed slightly larger fluctuations, about 1.21 times the Poisson expectation (see Experimental Procedures). It is known that mammalian M/T cell responses are highly variable (Bhalla and Bower, 1997; Chaput and Holley, 1985). To our knowledge, such variability has not been separated into trial-to-trial fluctuations in anesthetized animals and variability in underlying respiration tuning properties of M/T cells. It is the latter form of variability that affects the current analysis (see Discussion). We added this 1.21-times-Poisson noise estimate to our inferred air and odor inputs, and used a Monte Carlo procedure to simulate the distribution of chi-square values that would be observed if the model were true (Experimental Procedures). We then compared the actual chi-square value (obtained from fitting the experimental data) to this simulated distribution of chi-square values (obtained from the Monte Carlo procedure). If the actual value lies near the mean of the simulated distribution, it is very likely that the model is true. Using this procedure, we found that data from 80% (33/41) of our experiments were within the 99.9% boundary and 54% (22/41) were within the 95% boundary of the simulated chi-square scores (Figure 6). Since we have been conservative in our noise estimate (which is set at just 1.2 times the minimum possible level) it is appropriate to use the 99.9% cutoff rather than the overly stringent 95% cutoff (Press and Teukolsky, 1992) in selecting cells that are “well fit” by our model.

### Applicability of Model to Single-Odor Concentration Series

The above model should also hold for cases where a single odor is presented to the cell and its concentration is increased. The model predicts that an increase in the concentration of a single odor should lead to observations consistent with a single-odor input strength function growing in size. For example, for most simple input strength functions, one should observe the excitatory or inhibitory components of a response growing in amplitude and possibly in width.

**Figure 7:**
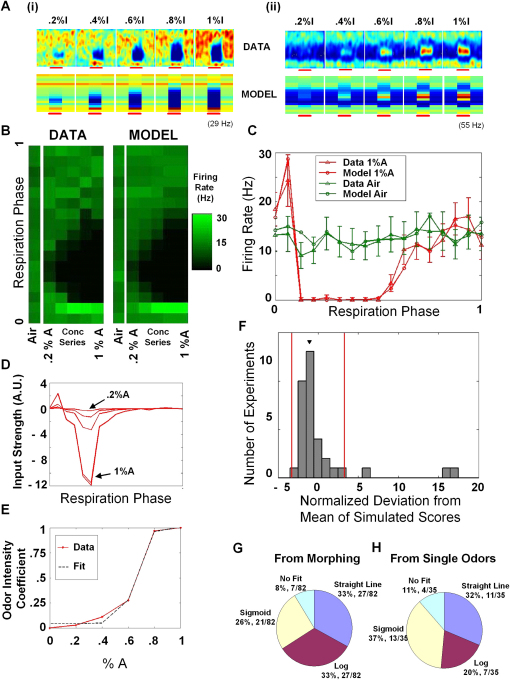
The Model in the Single-Odor Case (A) Two examples of the effect of increasing the concentration of a single odor on a cell's response, comparing data and model. A(i) is a primarily inhibitory response and A(ii) is primarily excitatory. Here the effect is an increase in the width and/or amplitude of the response. This contrasts with the shift in the tuning pattern seen in some of the mixture results. Example A(i) is explored in detail in the rest of the figure. (B) Comparing the entire concentration series and the average air period for the data and model for the cell shown in A(i), as in Figure 5B. (C) The 1% odor case and the air period overlaid for odor and model. Error bars indicate SEM. (D) The input strength functions for the odor at different concentrations. The large inhibitory component is evident, and the asymmetry in its shape explains why the inhibitory “gap” in the data increases in one direction more than the other (toward later respiration phases). (E) The plot of the coefficients against the externally applied odor concentration is shown overlaid with the best fit; in this case, a sigmoid. (F) The population analysis (akin to Figure 6), showing that 91% (32/35) of the experiments validate the model. Arrowhead indicates the bin in which example A(i) belongs. Vertical red lines are at ±3.29. (G and H) Distributions of coefficient plots across straight line, log, and sigmoid categories for the morph and single-odor experiments, respectively. These two distributions were not different as shown by a chi-square test (p < 0.05).

We performed these concentration series experiments on 24 cells (11 cells from the previous set, with two odor concentration series each, and 13 new cells that responded to only one odor and thus have one concentration series each, for a total of 35 series). Two examples of such experiments are shown in Figures 7A(i) and 7A(ii), with one primarily inhibitory and the other primarily excitatory. These examples illustrate the key prediction of the response, i.e., the increase in the amplitude or width of the responsive region but no shift in phase. The first example is further explored in detail in Figures 7B–7E. In Figure 7B the data and the prediction from the model are compared for all the concentrations and the air, as in Figure 5B. Figure 7C shows the firing rates as a function of respiration phase for the air period and the odor period for the 1% odor case. The data and the model curves are overlaid. In Figure 5D we show the underlying input strength functions with a strong inhibitory component. One can see from the asymmetry of this inhibitory component why the inhibitory “gap” increases more rapidly in one direction (toward the later respiration phases). Also, increasing this odor's concentration did not proportionately increase its effect on the cell, as is seen in the plot of the odor intensity coefficients in Figure 7E.

Applying the same model validation to this data as to the mixture data, we obtained the histogram in Figure 7F. Here, 91% (32/35) of the concentration series were within the 99.9% boundary and 63% (22/35) were within the 95% boundary of the chi-square scores expected if the model were true.

### Revisiting the Attractor Question

In the above sections we have shown that our model of M/T cell responses was able to encapsulate many of the encoding properties of these cells, and was quite accurate in describing how these responses changed with mixtures of odors. A key prediction of the model is that the contribution of each odor to the final output of each cell is represented in its odor intensity coefficient. This odor intensity coefficient is therefore a good measure of how much each cell represents one odor or another. This makes it a good variable to track over the morph sequence. Strong attractor dynamics would predict that the odor intensity coefficient should change abruptly through the morph sequence.

We obtained several kinds of curves when we plotted the odor intensity coefficient against odor proportion (Figure 5E and Figure 7E). As in Figure 3, we characterized the responses in terms of the best fit to straight lines, logarithms, and sigmoids (p < 0.01 and best explained variance by the F statistic). The distribution of responses is shown in Figure 7G (82 odor intensity coefficient plots, two for each of the 41 morph sequences). As before, we observed cells belonging to all categories. In particular, sigmoid responses characteristic of attractor dynamics were indeed seen, but accounted for only 26% of responses. Also, as mentioned earlier, there was no difference between the naive and familiarized groups (chi-square test, p < 0.05, Table S1).

We tested whether this broad distribution of odor morph responses was an inherent property of M/T cells. We did so by generating odor intensity coefficient curves from the experiments that involved only concentration series with a single odor (Figure 7E). The distribution of cellular responses is shown in Figure 7H. Again, we found that curves for odor intensity coefficients were distributed between straight lines, logarithms, and sigmoids. We performed a chi-square test between the distributions in Figure 7G and 7H, which showed that they were not different from each other (p < 0.05). Thus, even the relatively steep sigmoid transitions of odor intensity coefficients in the morph sequences were also seen in single-odor cases. There was no tendency for any of the four different odors to have a predominance of any category (chi-square test, p < 0.05).

As we discuss below, this suggests that all the properties shown by the cells in the odor-mixing experiments, including the fraction of sigmoidal transitions, can be inferred from the cases where single odors were presented separately, and may not require attractor dynamics.

### Direct Demonstration of Additivity

**Figure 8:**
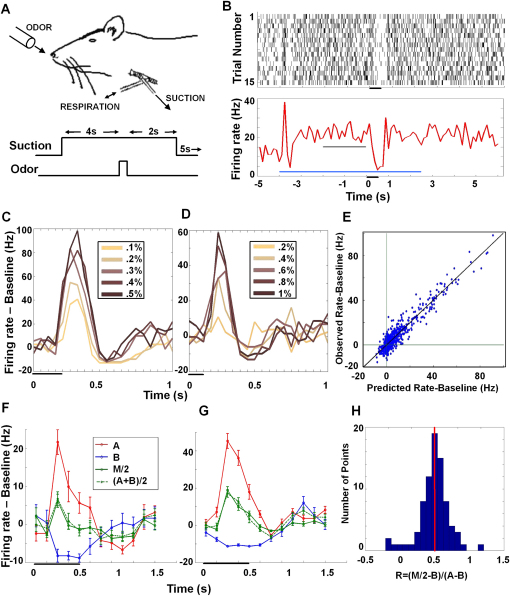
Validation of Scaling and Additivity (A) Diagram showing the double tracheotomy preparation and the odor presentation protocol. As earlier, an air stream was always blowing at the rat's nose and this was switched to an air + odor stream in the period shown (100, 200, or 500 ms). (B) Raster showing a cell with a flat baseline responding to a 500 ms odor pulse. Below is the PSTH of same cell binned at 125 ms. Black bar: odor duration; blue bar: suction duration; gray bar: 2 s period used for determining baseline firing rate. Both transient and stable firing rate changes from suction alone can be seen. (C and D) Two examples of cells showing the same-shaped response scaling in amplitude with odor concentration. Shown are 200 ms and 100 ms odor pulses [(C) and (D), respectively]; spikes are binned at 50 ms, and odor was iso-amyl acetate in both. Error bars indicating SEM have been removed for clarity. (E) Predicted versus observed firing rate; baseline is plotted for the 12/17 cells that were consistent with a single-shape scaling (Q value > 0.001). (F and G) Two examples of cells that showed that the response to the mixture was a direct summation of the responses to the components. A and B are the components and M is the mixture. The dashed green line shows what the mixture would be if there were perfect addition. The odor pairs in (F) and (G) are iso-amyl acetate/(+) limonene and methyl amyl ketone/iso-amyl acetate, respectively. Error bars indicate SEM. (H) Histogram of the values of R. Perfect addition would cause these values to be 0.5 (red line).

Our odorant and mixture representation model is complex because the respiration cycle introduces respiration phase dependencies. To directly test the core assumptions of the model, we eliminated respiration dependence. We did so using a double-tracheotomized preparation where air/odorant intake was continuous. In each of the 10–15 trials, we presented odor in the manner shown in Figure 8B. We found that cells were no longer respiration-tuned in these experiments and had a flat baseline, while they still responded in a time-dependent fashion to odors.

We tested whether the firing rate curves scaled in size while preserving their shape with increasing odor concentration. As seen in Figures 8C and 8D, this was indeed the case, and was true for a variety of odor pulse durations and concentration scales. To confirm this scaling rigorously, we fit all data in a given concentration series by a single curve varying only in amplitude. Predictions from this fit were consistent with the measured data for 12 out of 17 cells, as shown in Figure 8E.

We asked if the response to a 0.5% + 0.5% mixture of two odors eliciting different responses (henceforth, M) was the same as the sum of the responses to two individual odorants at 0.5% concentration (henceforth, A and B). We were able to record 15 neurons that responded to two odorants with this protocol. We found that the mixture M was well predicted by simply adding the individual responses A and B (M = A + B). Graphically, this can be interpreted as the curve M/2 lying halfway between the curves A and B [M/2 = (A+B)/2]. This is seen to be the case in Figures 8F and 8G. To quantify this data, we took all measured points where A and B were well separated and computed the ratio R = (M/2 − B)/(A − B). This ratio should be 0.5 if M is perfectly predicted by the model. We see that measured values of R indeed cluster around 0.5. The mean of the distribution is 0.49 ± 0.03, and 60 out of the 80 points lie within 2 standard errors from the value 0.5. (Figure 8H).

In summary, we find that most OB neurons respond to odor mixtures as a weighted sum of individual odor responses rather than as distinct attractor states. This is an economical encoding scheme for a stimulus modality rich in complex mixtures.

## Discussion

We have characterized M/T cell responses in the dimensions of odor identity and intensity, and we have explored responses in both dimensions through the use of odor combinations. We find that responses to mixtures morph smoothly between single odor responses; this trend is inconsistent with models of strong attractor dynamics occurring in the OB. We show that over a wide range of mixtures and concentrations, M/T cell responses can be described by a model of input strength functions acting on a cell, where different odorant contributions combine additively.

### Is the Olfactory Bulb an Attractor Network?

An attractor network would be expected to exhibit abrupt transitions upon presentation of morph sequences of odors. Most of our experiments do not show abrupt transitions. The smooth transitions between single-odor representations are apparent in both the simple analysis and the model-based analysis. In about 30% of cases in the model-based analysis, we see sigmoid transitions that are relatively steep. However, these could be accounted for by the responses of the cells to the increasing concentration of a single odor, as seen in the single-odor concentration series data. As there was no elevation in occurrence of abrupt transitions over the single-odor case, we consider the rat OB free from strong attractor dynamics. Our data are mostly from neurons recorded one at a time. A strong case for attractors would require simultaneous recordings from many neurons to show coherent transitions in the population. However, as we show that even cells recorded one at a time mostly show smooth transitions, abrupt population transitions may be ruled out. Attractor dynamics are invoked to explain recognition of stored patterns or categorization of stimuli. To explain these phenomena in olfaction, one will thus have to look at other mechanisms and other brain regions, a likely candidate being the olfactory cortex (Haberly, 2001; Haberly and Bower, 1989; Rennaker et al., 2007; Zou et al., 2005).

### M/T Cell Encoding of Single Odors and Mixtures

In the process of addressing the attractor question, we have developed an encoding model for the representation of odors in single M/T cells. This model describes three things about these cells' responses: the representation of odor identity, the representation of odor intensity, and the summation of odors in odor mixtures.

The model states that the firing rate profile of a cell over the respiration cycle arises from an underlying input strength function specific to each odor. These functions have the interesting property of scaling multiplicatively with odor concentration and summing for different odor-air contributions. The multiplicative odor scaling terms, or odor intensity coefficients, are a measure of overall input strength received by the cell from an odor at a particular concentration.

There are two distinct types of saturation that occur in our experiment. First, the response to any given odor tends to saturate at high odor concentrations. This is accounted for in the model because the intensity coefficients can saturate even as concentration increases, as seen in Figure 7E. Second, regardless of odor concentration, the firing rate of a cell must remain between zero and some physiologically constrained maximum value. In our model, firing rates are obtained by a sigmoidal transformation, and so are naturally restricted between zero and maximal values. Finally, we have observed that at high odor concentrations the response can change qualitatively, varying over the course of odor presentation as seen in Figure S5. In these cases the model would fail to capture the observed behavior.

Our model is an economical phenomenological model, and may be a useful stepping stone on the way to a mechanistic explanation. We suggest that the primary mechanistic insight is the additivity of the different odor contributions at the level of M/T cell responses. We speculate that such additivity is more likely at the input stage, rather than through feedback via granule cells. This is because the observed simple additive responses do not show history dependence, which might have been expected if feedback were present. Instead we suggest that convergent odorant signals, possibly arising from receptor neuron and periglomerular cell inputs, contribute to additivity at the inputs.

### M/T Cells Synthesize Novel Representations to Odor Combinations

In the cases where two different odors elicit activity in a cell with peaks at different phases of the respiration cycle, we often observe this peak shifting through intermediate phases on presentation of odor mixtures (Figures 2A, 2B and 2E). Thus, the identity of the mixture (as encoded by phase position) is now different from either of the two primary components. Odor mixtures are known to be elemental (the components are recognizable) or configural (the mixture is qualitatively different from the components) to a degree depending on concentration ratios (Kay et al., 2005). Our observations of phase-position morphing provide neuron-level mechanisms for configural odor mixtures. Consider the activity of a given M/T cell that responds to two odors, A and B, where each odor has a peak of activity at a different phase of respiration (e.g., Figure 2). If the response to a mixture were a simple weighted sum of the peaks due to A and B, it would be an elemental response, because the individual odor identities, as encoded by peak phase, are retained. Additionally, if different neurons responded independently to A and B, they too might contribute to an elemental response. This is the kind of response seen at the glomerular level of the OB (Lin et al., 2006). A configural response, on the other hand, occurs when mixtures give different responses from either individual odor, which is what we sometimes observe (Figures 2A, 2B, and 2E) and now explain in terms of our model (Figure 5). Though multiple levels of processing seem to be involved, including receptor neurons (Duchamp-Viret et al., 2003) and the glomerular layer (Linster and Cleland, 2004), we suggest that this transformation from elemental to configural responses is one of the computational functions of the OB M/T cells.

### Comparisons with Previous Studies

On examining earlier studies of M/T cell responses to different odors and concentrations, we found that their data could also be explained in the framework of this model. This is despite the fact that the conditions of the experiments were often very different. Cang and Isaacson (2003) performed whole-cell recordings in rats and measured intracellular postsynaptic potentials in response to odor stimuli. They observed that EPSPs and IPSPs both grew multiplicatively in amplitude with odor concentration, which is consistent with our model.

Chalansonnet and Chaput (1998) showed that when odor concentrations were increased, cells did not change their respiration tuning for successive concentrations. This is consistent with our model's claim that increasing concentration only increases the amplitude and not the shape of an odor input strength function.

While our study is based on natural respiration, some of our findings are consistent with those from a study with controlled airflow using tracheotomized rats and artificial sniffs (Harrison and Scott, 1986). This study reported odor responses that consisted of both excitatory and inhibitory components. Furthermore, the amplitude of both components of the response increased with odor concentration, which is in agreement with our data and model.

In a study in hamsters (Meredith, 1986) and in salamanders (Hamilton and Kauer, 1989), the authors reported complex odor responses consisting of both excitation and inhibition, which changed with intensity in a similar manner as we found. As in our study, these groups observed different timing patterns of M/T cell activity for different odors.

Our results are not in agreement with those of Giraudet et al. (2002), who find that one component in a binary mixture usually dominates in M/T cell responses. This disagreement may arise because their analysis does not consider the components of a response saturating and going below zero firing rate, whereas our analysis does.

### Limitations of the Model

There were three main limitations of our model. First, when the respiration tuning of the cell varied from cycle to cycle over the duration of odor stimulus, the model was unable to explain the results. Second, we frequently observed a drift in baseline firing pattern and response to an odor over the duration of a morph sequence (∼3 min). This was larger than that accounted for by Poisson noise and may have been due to anesthesia level fluctuations. We chose not to include this as a separate term in the model to avoid further complexity, and instead incorporated it in our estimate of noise as explained in the Supplemental Material. Finally, most of the experiments that did not fit the model were due to too large a baseline drift. However, in two examples of a concentration series with an odor, there was inhibition that changed to excitation at one respiration phase, and in one example there was a large shift in a band of excitation. Neither of these rare cases could be explained by our model.

### Relevance in Awake Rats

It has been observed that respiration tuning exists in M/T cells in awake rats, with a baseline tuning pattern for air that can change on odor presentation (Bhalla and Bower, 1997). Further, in awake rat recordings from piriform cortex, respiration-locked firing also exists and can be different for different odors (N. Uchida and Z.F. Mainen, 2006, Soc. Neurosci., abstract).

Thus, the basic property of respiration-phase tuned odor-specific responses is common to awake and anesthetized rats. We therefore predict that our model of M/T cell encoding of odors will also be applicable to awake animals.

## Experimental Procedures

We used standard extracellular single-unit recording techniques for our experiments. These methods are very similar to those used in two earlier studies from the lab (Deshmukh and Bhalla, 2003; Rajan et al., 2006). They are described in detail in the Supplementary Material. Briefly, female wistar rats (200–350 g) were anesthetized with xylazine (10 mg/kg) and ketamine (100 mg/kg), and anesthesia was maintained with thiopental. Only females were used since we could not induce complete surgical anesthesia to our satisfaction in males. Respiration was monitored by placing a thermocouple in the nostril.

Recordings were done with gold-plated tetrodes that were lowered from the dorsal surface of the bulb to the mitral cell layer. This we identified by the distinctive high-amplitude and respiration-locked multiunit activity. In a few cases we lesioned at the electrode tip and confirmed its placement in the mitral cell layer by sectioning and staining. Signals were amplified (10,000×) and band-pass filtered (300–6000 Hz), and triggered waveforms were digitized and stored at 32 kHz. Single-unit data were extracted by clustering using MClust (A.D. Redish; http://www.cbc.umn.edu/∼redish/mclust/).

Cells were classified as responsive to an odor using Student's t test and MANOVA. Cells responding differently to two odors were used in the morph experiments. Cells with very large changes in baseline (air period) firing rates over a morph sequence/concentration series were excluded from the study.

Odors were delivered using a computer-controlled air dilution olfactometer based on designs described earlier (Deshmukh and Bhalla, 2003; Slotnick and Nigrosh, 1974).

### Familiarization to Odors

One group of rats (n = 14) was familiarized to two odors (iso-amyl acetate and methyl amyl ketone) by a classical-conditioning-like protocol. They were water deprived for 20 hr and given water with iso-amyl acetate mixed in it at a final concentration of 0.01% for 4 hr. They were also food deprived for 20 hr and food was introduced into the cages preceded 5 min earlier by a small piece of cloth moistened with 1% methyl amyl ketone, for 4 hr. The two odor exposures were separated in time by at least 2 hr. This procedure was repeated for 5–8 days.

### Calculating Air and Odor Response Functions

To estimate air and odor response functions, we defined two Δ*t* = 7 s time windows, one within the air period, 1 to 8 s before odor valve opening, and the other within the odor presentation period, 1 to 8 s after odor valve opening. The 1 s period immediately after valve opening was avoided, because there were delays in the odor traveling down the delivery tube and because our odor valve opening was not synchronized with respiration. For each window, we binned firing events into *N_R_* = 17 bins along the respiration phase axis. This produced two vectors: *v^0^_i_* (air response) and *v^1^_i_* (odor response), periodic over the respiration cycle *i* = 1, …, *N_R_*, in units of firing rate.

### Modeling the Response to Mixtures of Odors

We modeled our neuron as having a sigmoidal response to simple linear inputs (Hertz et al., 1991):

[1] $$v_{i}=v_{max}f\left(w_{i}^{0}+c^{A}w_{i}^{A}+c^{B}w_{i}^{B}-b\right).$$

Here, *v_i_* represents the firing rate of a neuron during respiration phase *i*, which can take some saturating value *v_max_*. The vectors *w^0^_i_*, *w^A^_i_*, and *w^B^_i_* represent inputs to the neurons due to air, odor A, and odor B, respectively. The latter two are multiplied by concentration-dependent coefficients *c^A^* and *c^B^*. Without loss of generality, the value of the coefficient at the maximum odorant concentration is set to 1.0.

The function *f* (.) is a sigmoid, defined such that *f* (−0.5) = 0.1, *f* (0) = 0.5, and *f* (+0.5) = 0.9:

[2] $$f\left(x\right)=\frac{e^{4.39x}}{1+e^{4.39x}}.$$

Finally, the quantity *b* sets the baseline firing rate of the neuron in the absence of any inputs. Note the following dependencies: *v_max_* and *b* are fixed for any given neuron; the vectors *w_i_* are functions of respiration phase alone, but are concentration independent; and the coefficients *c* are functions of concentration alone, but are phase independent. These features strongly restrict the space of possible responses to mixed odors. In effect, we are claiming that the response to any mixture of odors is completely determined by the response to the individual components.

### Parameter Fitting

A typical morphing experiment involves *N_mix_* = 6 presentations of odors A and B, in the following proportions: [1.0A 0.0B], [0.8A 0.2B], [0.6A 0.4B], [0.4A 0.6B], [0.2A 0.8B], and [0.0A 1.0B]. For each such measurement, we obtained the air and odor responses *v^0^_i_* and *v^1^_i_*. Since the six air responses were not independent, we averaged them into a single vector $\langle v_{i}^{0}\rangle$. This gives:

$$N_{R}\left(N_{mix}+1\right)=119\text{datapoints}.$$

We fit these data to the neural model defined above. The concentration-dependent coefficients were defined such that *c^A^* = 1.0 and *c^B^* = 0.0 for pure A, and *c^A^* = 0.0 and *c^B^* = 1.0 for pure B, with their values for the four intermediate mixtures left as free parameters. Since the baseline *b* could not be determined independent of the vector *w^0^_i_* (this would require varying the “strength” of the air stimulus), this constant was absorbed into *w^0^_i_*. Adding in *v_max_*, *w^A^_i_*, and *w^B^_i_*, this resulted in

$$1+2\left(N_{mix}-2\right)+3\left(N_{R}\right)=60\text{parameters}.$$

This is a highly constrained fit, involving 59 degrees of freedom (d.f.). That is, if we use the first 60 datapoints to calculate the parameters, we claim that the remaining 59 datapoints will be completely determined. [Note that, in the single-odor case, we fit *N_R_* × *N_mix_* = 102 datapoints using 1 + (*N_mix_* − 2) + 2(*N_R_*) = 39 parameters, corresponding to 63 d.f.]

The model was initialized with suitable parameter estimates based on the response to pure odors, and the system was run to minimize the χ^2^ score defined in Equation 1 in the Supplementary Material. The minimization was performed in MATLAB (Mathworks), using the *fminsearch* function. This procedure was carried out for each independent morphing experiment.

### Estimating Significance of the Fit

We estimated the significance of our fit using a Monte Carlo technique (Press and Teukolsky, 1992). The χ^2^ statistic has a well-defined distribution for linear models. However, our model involves a sigmoidal nonlinearity, so we must be careful in estimating the background distribution of χ^2^ values against which to test the significance of the fit. For each morphing experiment, we proceeded as follows. Beginning with the best-fit predictions for the six odor presentations plus air, we generated a “fake data set” by adding Gaussian noise to each datapoint, with variance equal to α*_eff_* times the Poisson estimate (see the Supplementary Material). We then fitted parameters to this simulated data set, exactly as described above. This procedure was repeated for 50 trials, and the resulting parameters, as well as the resulting χ^2^ values, were recorded for each trial. This procedure allowed us to estimate the mean and variance of χ^2^ values, *assuming that the model is true, and that we understand noise sources*. We were therefore able to estimate the significance of our fit in terms of the p value: the fraction of times the simulated χ^2^ showed a greater deviation from its mean value than the actual χ^2^. If this number is close to unity, we can be confident that the model explains the observations without being overdetermined (χ^2^ too large) or underdetermined (χ^2^ too small); in practice, we can settle for a p value as low as 1e−3 or above, since a *wrong* model will typically produce a much lower value (Press and Teukolsky, 1992). This corresponds to ±3.29 standard deviations of a Gaussian, which defines the boundaries in Figure 6 and Figure 7F.

### Curve Fitting

For the simple analysis (Figure 3), shifting band responses and buildup responses were categorized by eye. Firing properties were quantified (see Supplementary Material) and plotted. These plots were fit to a straight line, a log, and a sigmoid. Fits with p < 0.01 were considered significant, and each fit was assigned to the category with the highest explained variance. The explained variance was measured with the F statistic, which is corrected for the different d.f. (d.f. = 2 for straight line and log fits and d.f. = 4 for sigmoid fits).

## Supplemental Data

Document S1. Supplemental Experimental Procedures, References, Figures, and Table
