# Supplementary material for: Odor Representations in the Rat Olfactory Bulb Change Smoothly with Morphing Stimuli
Source: Neuron. 2008 Feb 28;57(4):571–85. doi: 10.1016/j.neuron.2008.01.008 (PMC2258318; doi:10.1016/j.neuron.2008.01.008)
Supplement: Document S1. Supplemental Experimental Procedures, References, Figures, and Table [file mmc1.pdf]

Neuron, volume 57

Supplemental Data

Odor Representations in the Rat Olfactory Bulb Change Smoothly with Morphing Stimuli

Adil G. Khan, Mukund Thattai, and Upinder S. Bhalla

Details of experimental procedures

Animals and Surgical Procedures

All experimental procedures were approved by the National Centre for Biological Sciences institutional animal ethics committee, in accordance with the guidelines of the Government of India and equivalent guidelines of the Society for Neuroscience. Female Wistar rats, 3-4.5 months old and weighing 200-350 g were anesthetized with a mixture of ketamine (100 mg/kg; Themis Chemicals, Mumbai, India) and xylazine (10 mg/kg; Indian Immunologicals, Hyderabad, India). Only females were used since we could not induce complete surgical anesthesia to our satisfaction in males. Cells from 27 rats were used in this study. A surgical plane of anesthesia was maintained with 6.25 mg thiopental sodium (Abbott Laboratories, Bombay, India) injected IP at ~40 min intervals for the first 2-3 hours and longer intervals later depending on the anesthesia level. The surgical plane of anesthesia was judged by lack of foot withdrawal reflex. The rectal temperature of the rat was maintained at ~35°C with an infrared lamp. The animal was placed in a stereotaxic apparatus and an exposure of ~1.5 mm diameter was drilled over the left OB using a handheld drill (Fine Science Tools, North Vancouver, Canada) +8 mm anteroposterior and 1 mm lateral from bregma. The dura was removed with a pair of fine forceps (Dumont, Montignez, Switzerland). Respiration was monitored with a fine (<200 μm), insulated thermocouple (Physitemp, USA) inserted in the nostril. The thermocouple signal was amplified 1000-fold and stored on the computer after digitization at 320 Hz.

Recordings

Extracellular single unit recordings were made from the OB using tetrodes constructed from 13μm nichrome wire with Formvar insulation (Kanthal, Palm Coast, USA). The electrode tips were gold plated with noncyanide gold plating solution (SIFCO Selective Plating, Cleveland, USA) to final tip impedance of 150-250 kΩ at 1 kHz. 2-6 tetrodes were independently lowered into the OB until the mitral cell layer was reached. This was characterized by very high and distinctive background activity modulated by respiration. The tetrodes were lowered further till clear single unit activity could be discerned on an oscilloscope and through audio playback. A few recording sites were electrolytically lesioned after the experiment and brains were cryosectioned and stained with cresyl violet to confirm the presence of lesions in the mitral cell body layer.

The signals were pre-amplified 10 fold and further amplified 1000-fold and band-pass filtered between 300 Hz and 6 kHz on custom made amplifiers. The amplified signals were digitized at 32 kHz on a DAP3200e data acquisition card (Microstar Laboratories, Bellevue, USA) controlled with custom-written software, and stored as triggered waveforms on the computer.

Single unit data was extracted by clustering using MClust (A. D. Redish; http://www.cbc.umn.edu/~redish/mclust/). Well-defined cluster boundaries and the presence of a distinct refractory period as seen in the inter-spike interval histogram for the neuron were used as criteria for well isolated clusters.

Respiration rasters were created as described in Figure 1C. Each spike was plotted on a 2D plane with phase of respiration on the Y axis and the time on the X axis, with respect to the opening of the odor valve. Each odor or odor mixture was presented 5 times for 8 seconds each followed by 22 seconds of air. The respiration rasters included all 5 presentations superimposed and these were smoothed (Gaussian kernel of standard deviation 1.67s on time axis and .11 respiration phase on respiration phase axis) and color coded only for visualization.

Cells were classified as responsive to an odor if they either changed their firing rate during the odor period compared to the air period (t-test, $p < .01$), or they changed their respiration tuning (MANOVA between 6 respiration bins in air and odor periods, $p<.05$). Cells were included in the morph experiments only if they had two different responses to two odors. This was determined by a MANOVA between 6 respiration bins in the two odor periods. If overall firing rates were different between any two groups being compared by MANOVA (t-test $p<.01$), the respiration bins were normalized to the largest value bin to avoid false positives. Cells with very large changes in baseline (air period) firing rates over a morph sequence/concentration series were excluded from the study. Total number of cells indicated comprises cells which were tested with at least 3 odors in the naïve rats and the 2 familiar odors in the familiarized rats.

We also performed experiments (n=17 morphs, data not shown) where we did a morph experiment on a cell where only one of the two odors elicited a response. We found that these were explained by fitting them as a single odor concentration series. In other words, the second odor has no detectable effect on a morph sequence if it elicits no response on the cell in the first place.

Tracheotomized rat experiments

For the tracheotomized rat experiments, a double tracheotomy was done and tubes were inserted into the trachea in both directions as shown in figure 8A and tied in place. The rat breathed through one tube from which respiration was also recorded, and the other end was connected to a vacuum source. We recorded from 24 rats in this set of experiments.

We applied a suction 4s before and till 2s after the odor delivery at a flow rate of 250-300ml/min. The air suction alone would often cause both transient and stable firing rate changes. We confirmed that the cells in this preparation were no longer respiration tuned as above. In the concentration series experiments, the conditions used were: .2s odor pulse, .1 to .5% odor (n=10 cells), .1s pulse, .2 to 1% (n=4 cells), .5s pulse, .2 to 1% (n=3 cells). Each odor was presented for 15 trials, except for the .2s pulse cases which were 10 trials each.

The PSTH's were constructed by aligning each trial with the odor valve opening and binning at 50ms for the concentration series and 125ms for the mixture experiments. We

removed the first trial from concentration series experiments and first two trials from mixture experiments from each session because they sometimes showed a difference in the response. The average of a 2s window just before the opening of the odor valve was used to calculate the baseline firing rate as well as the noise in baseline firing. This was subtracted from the firing rate of the odor response period, which extended till 1s for concentration series and 1.5s for mixture experiments after odor valve on.

For analyzing the concentration series in the tracheotomized cases, we began with the 5 baseline-subtracted firing curves corresponding to the 5 values of odor concentration. Each curve was represented as a 20 point vector determined by binning 1s of firing data in 50ms intervals. To avoid the effects of zero-saturation, we only used those time bins in which the firing rate was significantly above zero (>1.5 standard deviations of baseline noise) at all concentration values. We used singular-value decomposition to fit all 5 curves to a model in which a single vector was scaled in amplitude alone, and obtained a chi-squared score quantifying deviations between the model's predictions and the observed data. The significance of this chi-squared statistic was then determined in the standard manner (Press and Teukolsky, 1992), in terms of a Q-value giving the probability that even a correct model would exhibit a deviation larger than that observed. In practice, models with $Q>0.001$ can be considered as being consistent with the observed data. We found that 9/17 cells had $Q>0.1$, 11/17 cells had $Q>0.01$, and 12/17 cells had $Q>0.001$. In Fig. 8E, we show the measured firing rates versus the model's predictions, pooling all the data from these 12 cells.
Further the average scaling coefficient changed 3.5 fold over the concentration series showing that the shape indeed does scale and that we were not comparing the same curves.

Odor Delivery

The following odors were used in these experiments: isoamyl acetate, methyl amyl ketone, 1,4-cineole and (+) limonene (Sigma-Aldrich, St. Louis, USA). Odor pulses were generated using a computer controlled air dilution olfactometer based on previous work (Deshmukh and Bhalla, 2003; Slotnick and Nigrosh, 1974) (Supplementary Figure 1). Saturated odor vapor was produced by bubbling nitrogen at 1 l/min measured by an acrylic block flow meter (Cole-Parmer, Illinois, USA) through liquid odor. The odor was immersed in glass beads to prevent aerosol formation. This was brought to 1% of saturated vapor through two stages of serial dilution with charcoal filtered and de-humidified air. A set of final valves directed either a filtered air stream or the odor stream toward the rats' nose. The odor valves were switched on 3 seconds before the final valves were switched, to eliminate transient high concentration odor puffs.
To generate binary odor mixtures, the output from two odor bubblers were mixed. The flow from each bubbler was adjusted to the values for each odor in the morph sequence, i.e. .2, .4, .6 and .8 l/min. Odors were directed to the exhaust for an initial 3s to allow equilibration, then the final valves switched the mixture to the rat. To generate a concentration series, one of the two odor bubblers was replaced with a blank.

Simple Analysis

We first assigned categories to the odor morphing changes in M/T cell response. Each cell's responses were assigned by eye to be either a shift along the respiration phase axis

of a band of excitation, or a change in firing in a certain respiration phase-range, or neither of these. To quantify the shifting bands we first binned the respiration raster into one bin for the odor period (+1 to +8 sec after odor valve opening as above) and 51 bins along the respiration phase axis (see Figure 3A). This was fit to a Gaussian. The peak of the Gaussian was taken to represent the position of the band. To quantify the firing rate buildup or decrease, we counted the total number of spikes fired over a respiration phase range during the same odor period. The phase range was determined by taking the pure odor condition with the most firing in that range and fitting it with a Gaussian as above. The boundaries of this firing band were decided either by visual inspection or by using the Gaussian fit as a guide with approximately 95% limits.

The number of spikes in the selected range was plotted against the concentration of the component odors on the x axis.

For the model-based analysis we used the odor coefficients on the y axis, and again used the concentration of odors on the x axis.

Quantifying noise in the observations

In order to test for uniformity of the response over time, we sub-divided each of the 17 bins into $N_T = 30$ parts along the time axis. We then calculated the mean and variance of the number of firing events in each such sub-bin. A neuron with a constant firing rate should produce a Poisson distribution of firing events, with the variance being equal to the mean. This is what we observed (Supplementary Fig. 2). This showed that over the course of any given air/odor period, firing rates depended only on respiration phase, not time. However, a complete experiment involved the presentation of several odor mixtures. Over this much longer time period, firing rates were seen to fluctuate slightly, as shown by the variation in the response of a single neuron to air alone. To quantify this variation, we selected $N_{neuron} = 12$ neurons that showed a particularly stable response to air over a complete experiment. For each neuron, we calculated the vector v^0_i over $N_{mix} =$ 6 different odor presentations, and calculated the variance of firing events over these 6 replicates. The resulting variance was slightly greater than expected from Poisson statistics. Assume that, for each data point, the observed variance is greater than the Poisson estimate by a factor α. This factor, which could vary from instance to instance but is drawn from some underlying distribution, has a quantifiable effect on any χ^2 calculation, as follows:

$$\chi^2_{true} \equiv \sum_i \frac{(x-x_i)^2}{\sigma_i^2} = \sum_i \frac{(v-v_i)^2}{\alpha_i v_i} = \langle 1/\alpha \rangle \sum_i \frac{(v-v_i)^2}{v_i} \equiv \langle 1/\alpha \rangle \chi^2_{nominal}, \qquad [1]$$

where we have assumed that α and x are uncorrelated. That is, for the purposes of a χ^2 test, the effective variance is increased over the Poisson estimate by an effective factor $\alpha_{eff} = \langle 1/\alpha \rangle^{-1} = 1.47$, calculated using $N_R \times N_{mix} \times N_{neuron} = 1248$ data points. Note that this is a very conservative noise level: the standard deviation is increased over Poisson by just $\sqrt{\alpha_{eff}} = 1.21$.

Supplementary References

Deshmukh, S. S., and Bhalla, U. S. (2003). Representation of odor habituation and timing in the hippocampus. J Neurosci *23*, 1903-1915.

Slotnick, B. M., and Nigrosh, B. J. (1974). Olfactory stimulus control evaluated in a small animal olfactometer. Percept Mot Skills *39*, 583-597.

Press, W., and Teukolsky, S. (1992). Numerical Recipes in C, Cambridge University Press).

Supplementary Figures and Legends

Supplementary Figure 1: Olfactometer

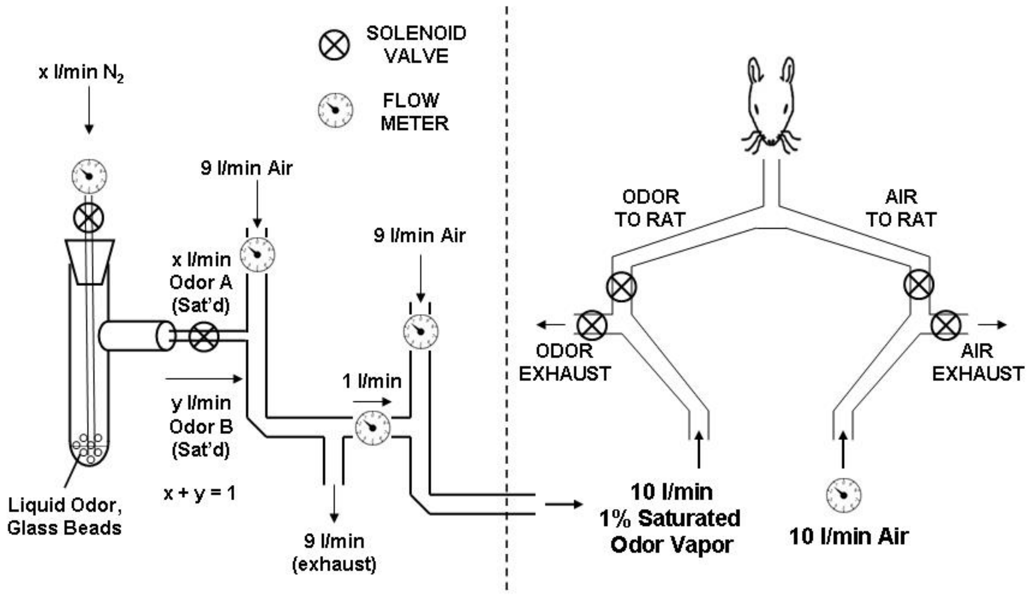

Figure 1: Olfactometer. The olfactometer consisted of bubblers which produced saturated vapor of the odors which was brought to 1% of saturated vapor by serial air dilution. Mixtures could be made by switching on two bubblers at a time. Using a set of final valves (right side) there was either always a stream of clean air blown at the rat's nose and the odor going to the exhaust, or when the valves were switched, the odor would be delivered to the rat and the air to the exhaust. In this way the odor(s) could be switched on 3 seconds before the delivery time, allowed to equilibrate while being sent to exhaust, and then presented as a step pulse by switching the valves.

Supplementary Figure 2: Noise is Poisson

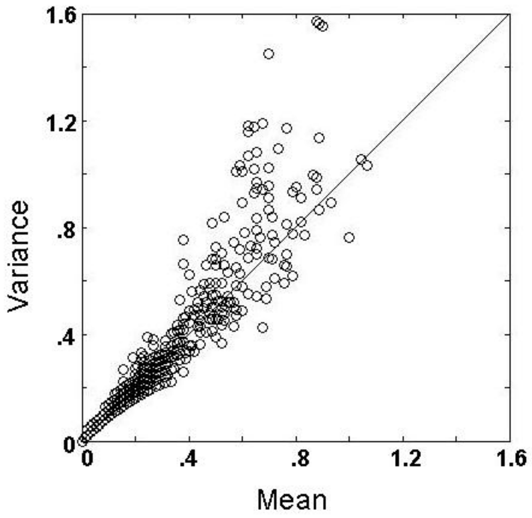

Figure 2: Noise is Poisson. The mean and variance are calculated for each bin as described in the text and plotted against each other. The points lie approximately along the diagonal, indicating the noise was largely Poisson in origin.

Supplementary Figure 3: 10X morph

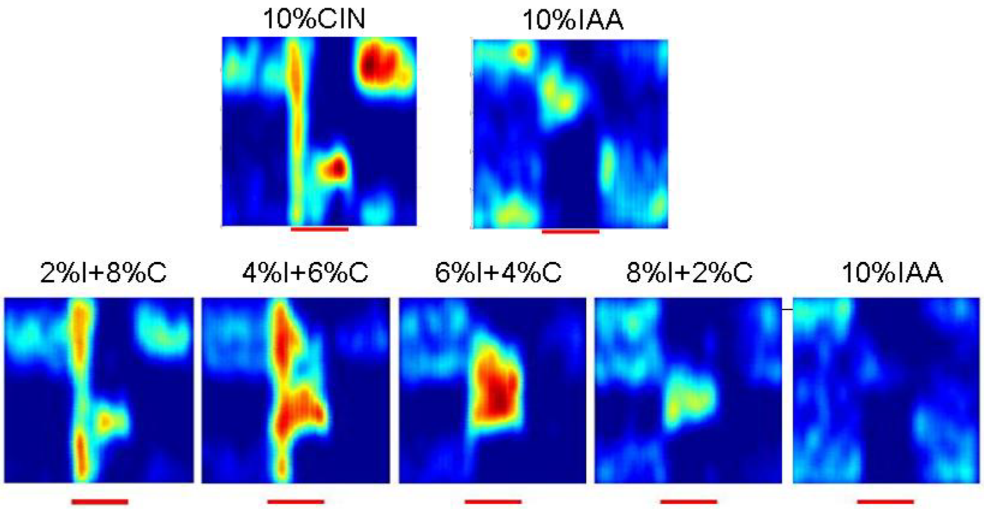

Figure 3: 10X Morph. The responses to 10% odor were typically not stable over the odor presentation period, but were more complicated. If the morph was performed at these higher concentration values, the phenomenon of responses passing through intermediate forms was still present.

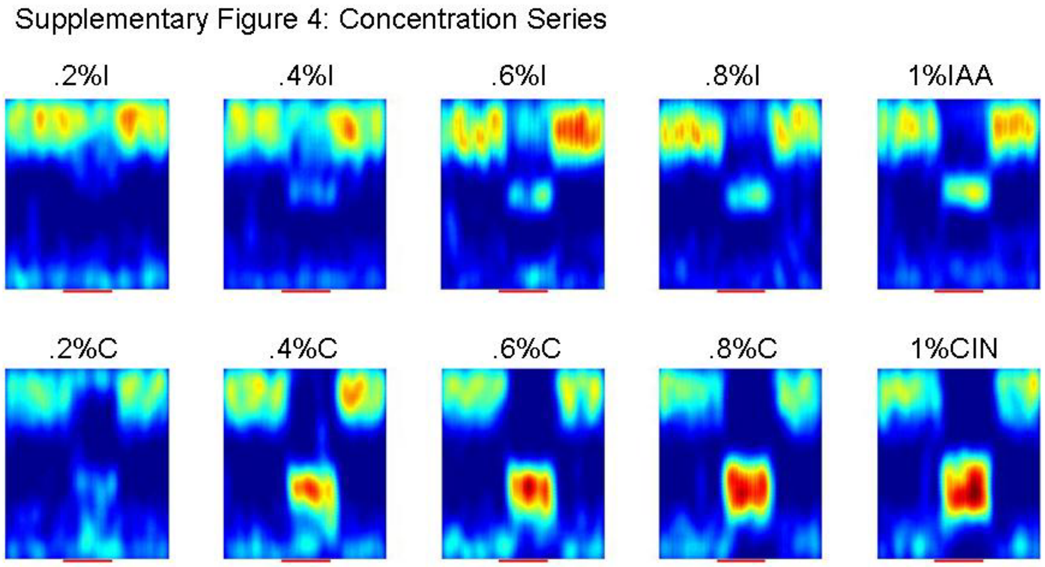

Figure 4: Examples of Concentration series: This is the same from Figure 5. The concentration series of the individual odors shows both excitatory and inhibitory components growing simultaneously. These may be compared with the prediction from the scaling Input Strength Functions in Figure 5D

Supplementary Figure 5: 10X concentration series

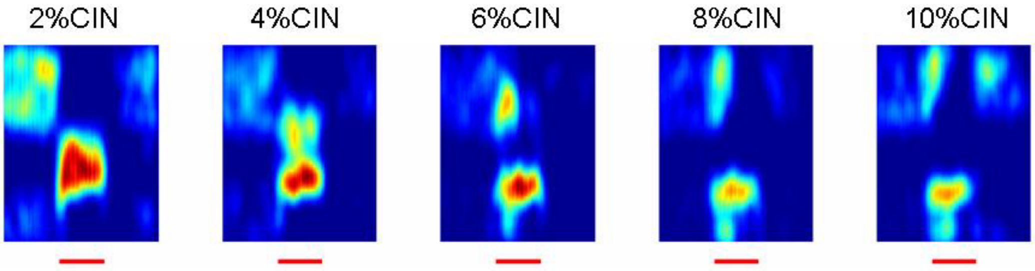

Figure 5: 10X concentration series: A concentration series going in steps of 2% up to 10%. This is the same cell from Figure 5. The transition from stable to complex responses over the odor period can be seen to be a smooth transition.

Supplementary Figure 6: Different bin sizes

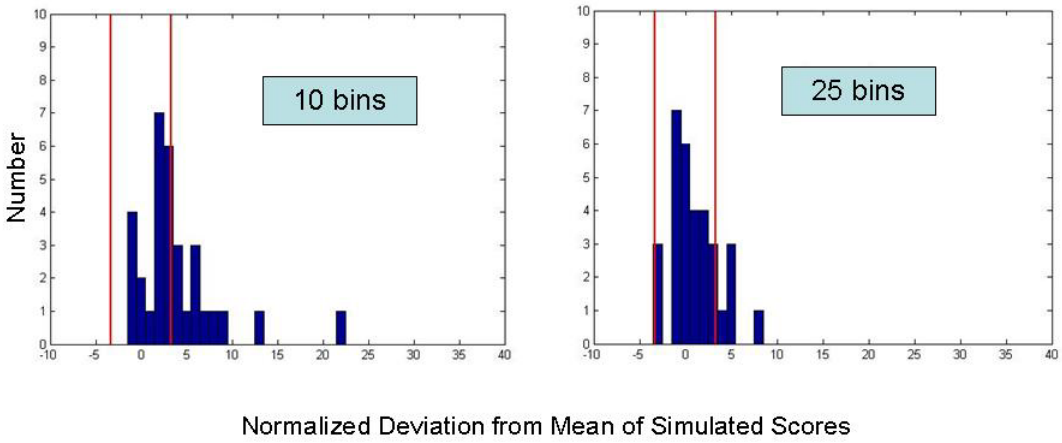

Figure 6: Model validation using different bin sizes for the two odor morphs. Using bin sizes of either 10 or 25 resulted in either less (10 bins) or the same number (25 bins) of morph experiments being within the acceptable cutoffs.

Supplementary Figure 7: 11-coefficient Fourier fits cannot capture sharp changes

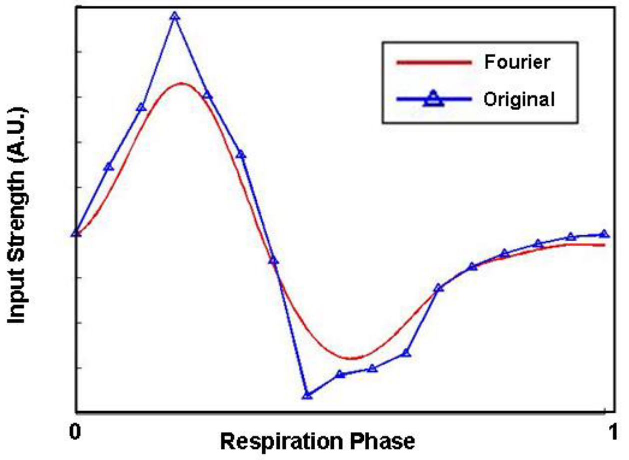

Figure 7: Fourier fits: When we tried to describe our input strength function curves with a curve described by 11 Fourier coefficients (11 parameters), we could not capture the sharp changes in the responses of the cells, thus justifying our use of a 17 point vector to describe these curves.

Supplementary Figure 8: No transient responses on odor onset

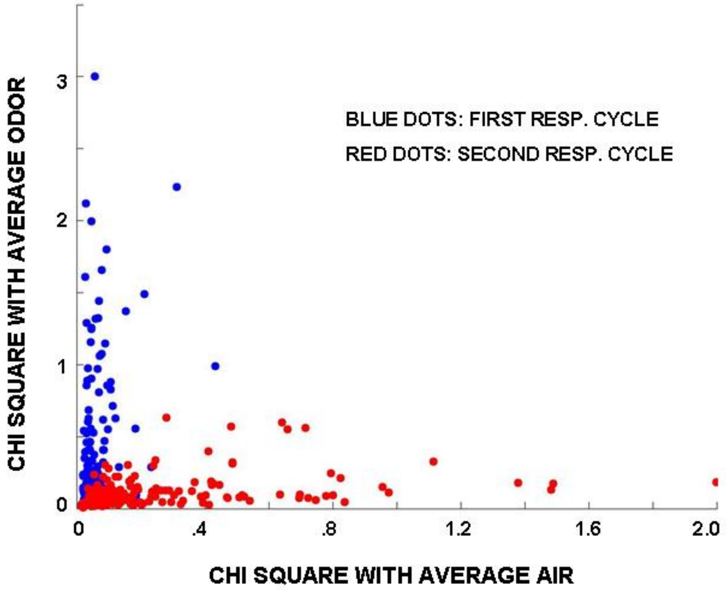

Figure 8: First 2 respiration cycles after odor valve opening: The first respiration cycle after odor valve opening was extracted for each of the odors/odor mixtures (with the 5 trials and averaged). This was compared with the average air response and the average odor response. We wished to determine if the first respiration cycle is an altogether new shape different from either of the two. We calculated the chi-square value (a measure of similarity of the two, larger values mean less similarity) for this curve with the average air and odor. These points were plotted on the figure shown (blue dots). It can be seen that these are almost always similar to the air response, suggesting that the odor has not yet reached (since our delay in the tubing was about the duration of one respiration cycle). The same was done for the second respiration cycle, and it can be seen in the figure (red dots) that these were predominantly similar to the average odor response and very different from the average air response. This suggests that there were not many significantly different transient responses in the first respiration cycle on odor arrival at the rat, and the cell immediately starts firing like its average response.

Supplementary Table 1: Distribution of morph experiments across categories compared between naïve and familiarized groups of rats. Both comparisons showed that for both simple and model based analysis, the distributions were not different (Chi square test, $p<.05$). Also we compare the distributions obtained by the different methods. These are also not different from each other by the same test.

**Simple Analysis**			
**Naïve**			
Linear	Log	Sig	None
6	4	2	7
**Familiarized**			
Linear	Log	Sig	None
7	6	1	1

**Model based Analysis**			
**Naïve**			
Linear	Log	Sigmoid	None
16	15	14	5
**Familiarized**			
Linear	Log	Sigmoid	None
11	12	7	2
